# Practical Notes on Diagnosis and Treatment

**Published:** 1913-04-12

**Authors:** 


### PRACTICAL NOTES ON DIAGNOSIS AND TREATMENT.

**Renal Calculus.**

Retraction of the testicle as a symptom of this condition is much more frequent in the child than the adult, as in the latter not only is the testicle heavier but the cremaster muscle is relatively feeble.

**Holidays.**

"Bear this in mind, that the world will use you up if you let it do so. And when you die before your time it will say—Ah! poor fellow, it is a pity he worked so hard!"—*Sir James Goodhart.*

**Ocular Paralysis in Migraine.**

An ocular paralysis is sometimes found to complicate migraine in young patients. The paresis disappears, but recurs with subsequent attacks of headache, and it is apt in time to become permanent.

**Paralysis Agitans.**

This is well known to be very resistant to treatment. The most likely method to prove helpful is a combination of Swedish movements, massage after a hot bath, and hyoscine $\frac{1}{100}$ grain thrice daily.

**Parotitis.**

This may complicate various abdominal conditions. It sometimes occurs after an attack of hæmatemesis, due, presumably, to a gastric ulcer. There may be much fever, and sometimes the inflamed gland suppurates. Occasionally salivary glands other than the parotid are affected.

**Atypical Graves's Disease.**

Dr. H. L. McKisack draws attention to cases in which palpitation, with perhaps flushing, tremor, and nervousness are the only symptoms, exophthalmos and goitre being almost or altogether absent. The true nature of such cases may readily be overlooked. Dr. McKisack advises a trial of *x*-ray treatment as having a fair prospect of success.—*British Medical Journal.*

**Chorea in Pregnancy.**

This almost invariably, when it occurs at all, occurs in first pregnancies. It is a condition by no means free from danger. Rest in bed, quiet, freedom from anxiety, and generous feeding are indicated; chloral hydrate and chloralamide are the most useful drugs. Sometimes gentle massage is helpful. Abortion is seldom necessary, but with failure of other treatment, loss of flesh, delirium, and continuing rapidity of pulse, it may be called for.

**Disseminated Sclerosis.**

The exhibition of this disease varies very widely in different cases, because the level of the distribution of the pathological lesion varies. In not a few cases the symptoms are confined to the lower limbs —the limbs become tired or numb after exertion, the gait is unsteady, and there may be difficulty with the bladder; and there are exaggerated knee- and heel-jerks, ankle clonus, and Babinski's sign. The diagnosis is certain, although nystagmus, slurred speech, and intention tremor are all absent.

**Food in Nephritis.**

According to von Noorden, while the reduction of albumen in the food to the lowest possible level is a wise proceeding in acute nephritis, it is a serious error in the chronic forms of the disease.

**Status Epilepticus.**

The recurring attacks in status epilepticus are best treated by hyoscine subcutaneously or chloral hydrate *per rectum.* Morphine will certainly check the seizures, but cannot be used without risk.

**The Pupil in Gastric Crises.**

In a tabetic patient the pupil during a gastric crisis may be dilated, presumably as a reflex consequence of the pain. This, of course, may help to mislead the physician in the recognition of the true nature of the gastric attack.

**Ophthalmia Neonatorum.**

The best prophylactic is a 2 per cent. solution of nitrate of silver. This should be used in every case in which there is a recent history of any vaginal discharge. The disadvantage is that the silver nitrate causes some degree of conjunctivitis.

**Leucodermia.**

Patches of leucodermia may sometimes be found on the neck in secondary syphilis. They are often inconspicuous and may not have attracted the patient's attention. In the case of a doubtful eruption such patches are of much diagnostic value.

**Myopia in Diabetes.**

In every case of myopia commencing in middle life a careful examination of the urine for sugar should be made. The association is not quite easy to explain, but the experience is undoubted that the development of myopia in an adult is sometimes the first evidence of diabetes.

**Epilepsy.**

When bromides do not succeed, their influence may be seconded by borax, or belladonna, or digitalis. Belladonna appears to be especially useful in children; digitalis when there is some degree of hysteria; and borax may be tried in patients who seem to have become so accustomed to the bromide that this has lost its influence.

**Acute Eczema.**

An attack of acute eczema, sometimes widely distributed, may follow exposure to cold. Calomel followed by a saline purge is indicated on general principles, but the special remedy called for is tartar emetic. Antimonial wine—say, $7\frac{1}{2}$ min.—should be given every two or three hours for six doses, and then continued thrice daily.

**Chronic Bronchitis.**

Dr. Charles Read on the basis of personal experience advises the persevering use of lozenges containing, each, heroin $\frac{1}{12}$ gr. and menthol $\frac{1}{10}$ gr. He takes one lozenge at bed-time, and, if cough or breathlessness is troublesome, a second one during the night. He is not conscious of anything of the nature of a heroin habit.—*British Medical Journal.*

